# Venoarterial PCO_2_-to-arteriovenous oxygen content difference ratio is a poor surrogate for anaerobic metabolism in hemodilution: an experimental study

**DOI:** 10.1186/s13613-017-0288-z

**Published:** 2017-06-12

**Authors:** Arnaldo Dubin, Gonzalo Ferrara, Vanina Siham Kanoore Edul, Enrique Martins, Héctor Saúl Canales, Carlos Canullán, Gastón Murias, Mario Omar Pozo, Elisa Estenssoro

**Affiliations:** Cátedra de Farmacología Aplicada, Facultad de Ciencias Médicas, Universidad Nacional de La Plata, Calle 42 Nº 577, 60 y 120, 1900 La Plata, Argentina

**Keywords:** Hemodilution, Hemorrhage, Anaerobic metabolism, Oxygen, Carbon dioxide, Respiratory quotient

## Abstract

**Background:**

The identification of anaerobic metabolism in critically ill patients is a challenging task. Observational studies have suggested that the ratio of venoarterial PCO_2_ (P_v–a_CO_2_) to arteriovenous oxygen content difference (C_a–v_O_2_) might be a good surrogate for respiratory quotient (RQ). Yet P_v–a_CO_2_/C_a–v_O_2_ might be increased by other factors, regardless of anaerobic metabolism. At present, comparisons between P_v–a_CO_2_/C_a–v_O_2_ and RQ have not been performed. We sought to compare these variables during stepwise hemorrhage and hemodilution. Since anemia predictably produces augmented P_v–a_CO_2_ and decreased C_a–v_O_2_, our hypothesis was that P_v–a_CO_2_/C_a–v_O_2_ might be an inadequate surrogate for RQ.

**Methods:**

This is a subanalysis of a previously published study. In anesthetized and mechanically ventilated sheep (*n* = 16), we compared the effects of progressive hemodilution and hemorrhage by means of expired gases analysis.

**Results:**

There were comparable reductions in oxygen consumption and increases in RQ in the last step of hemodilution and hemorrhage. The increase in P_v–a_CO_2_/C_a–v_O_2_ was higher in hemodilution than in hemorrhage (1.9 ± 0.2 to 10.0 ± 0.9 vs. 1.7 ± 0.2 to 2.5 ± 0.1, *P* < 0.0001). The increase in P_v–a_CO_2_ was lower in hemodilution (6 ± 0 to 10 ± 1 vs. 6 ± 0 to 17 ± 1 mmHg, *P* < 0.0001). Venoarterial CO_2_ content difference and C_a–v_O_2_ decreased in hemodilution and increased in hemorrhage (2.6 ± 0.3 to 1.2 ± 0.1 vs. 2.8 ± 0.2 to 6.9 ± 0.5, and 3.4 ± 0.3 to 1.0 ± 0.3 vs. 3.6 ± 0.3 to 6.8 ± 0.3 mL/dL, *P* < 0.0001 for both). In hemodilution, P_v–a_CO_2_/C_a–v_O_2_ increased before the fall in oxygen consumption and the increase in RQ. P_v–a_CO_2_/C_a–v_O_2_ was strongly correlated with Hb (*R* ^2^ = 0.79, *P* < 0.00001) and moderately with RQ (*R* ^2^ = 0.41, *P* < 0.0001). A multiple linear regression model found Hb, RQ, base excess, and mixed venous oxygen saturation and PCO_2_ as P_v–a_CO_2_/C_a–v_O_2_ determinants (adjusted *R* ^2^ = 0.86, *P* < 0.000001).

**Conclusions:**

In hemodilution, P_v–a_CO_2_/C_a–v_O_2_ was considerably increased, irrespective of the presence of anaerobic metabolism. P_v–a_CO_2_/C_a–v_O_2_ is a complex variable, which depends on several factors. As such, it was a misleading indicator of anaerobic metabolism in hemodilution.

## Background

The identification of anaerobic metabolism in critically ill patients can be elusive. Hyperlactatemia, central venous oxygen saturation, or isolated values of oxygen transport and consumption (DO_2_ and VO_2_) are frequently misleading indicators of tissue hypoxia. In contrast, the acute increase in respiratory quotient (RQ) is an excellent marker of ongoing anaerobic metabolism in exercise [1] and oxygen supply dependency conditions [2, 3]. In both circumstances, there is an excess of carbon dioxide production (VCO_2_) compared to VO_2_. This is the result of anaerobic VCO_2_, which arises from the bicarbonate buffering of anaerobically generated protons [1]. The proper measurement of RQ, however, requires analysis of expired gases. This monitoring is not usually available in the critical care setting. Recently, some observational studies have suggested that the ratio of venoarterial PCO_2_ (P_v–a_CO_2_) to arteriovenous oxygen content difference (C_a–v_O_2_) might be a good surrogate for RQ. Accordingly, high P_v–a_CO_2_/C_a–v_O_2_ has been associated with hyperlactatemia [4], decreased lactate clearance [5, 6], oxygen supply dependency [7, 8], and worse outcome of critically ill patients [4]. Nevertheless, P_v–a_CO_2_/C_a–v_O_2_ might theoretically be increased by several other factors irrespective of the presence of anaerobic metabolism. Moreover, comparisons between P_v–a_CO_2_/C_a–v_O_2_ and RQ have not been performed yet.

Given the increasing number of publications about the P_v–a_CO_2_/C_a–v_O_2_ and the lack of an adequate validation, further research is needed. This study was derived from a secondary subanalysis of a previous publication that sought to determine the relationship among oxygen transport, microvascular perfusion, and tissue CO_2_ in ischemic and anemic hypoxia [9]. The present investigation was focused on the behavior of P_v–a_CO_2_/C_a–v_O_2_ and its determinants, as well as its relationship with RQ, during stepwise hemorrhage and hemodilution. Since a progressive hemodilution, which does not compromise aerobic metabolism, will predictably result in increased P_v–a_CO_2_ [10] and decreased C_a–v_O_2_ [11], our hypothesis was that P_v–a_CO_2_/C_a–v_O_2_ might be an inadequate surrogate for RQ in isovolemic anemia.

## Methods

### Anesthesia and ventilation

Sixteen sheep (20 ± 2 kg, mean ± SEM) were anesthetized with 30 mg/kg of sodium pentobarbital, intubated, and mechanically ventilated with a Servo Ventilator 900C (Siemens-Elema AB, Solna, Sweden) with a tidal volume of 15 mL/kg, a FiO_2_ of 0.21 and a positive end-expiratory pressure of 6 cm H_2_O. The initial respiratory rate was set to keep the arterial PCO_2_ between 35 and 40 mmHg. This respiratory setting was maintained during the rest of the experiment. Neuromuscular blockade was performed with pancuronium bromide (0.06 mg/kg). Additional pentobarbital boluses (1 mg/kg) were administered hourly and when clinical signs of inadequate depth of anesthesia were evident. Analgesia was provided by fentanyl as a bolus of 2 µg/kg, followed by 1 µg/h/kg. These drugs were administered intravenously.

### Surgical preparation

A 7.5-French Swan-Ganz standard thermodilution pulmonary artery catheter (Edwards Life Sciences, Irvine, CA, USA) was inserted through an introducer in the right external jugular vein to obtain mixed venous samples; its side port was used to administer fluids and drugs. Catheters were placed in the descending aorta via the left femoral artery to measure blood pressure, perform the bleeding, and obtain blood samples, and in the inferior vena cava to infuse fluids during isovolemic hemodilution.

### Measurements and derived calculations

VO_2_, VCO_2_, and RQ were measured by analysis of expired gases (MedGraphics CPX Ultima, Medical Graphics Corporation, St. Paul, MN). VO_2_ and VCO_2_ were adjusted to body weight.

Arterial and mixed venous PO_2_, PCO_2_, pH, Hb, and O_2_ saturation were measured with a blood gas analyzer and a co-oximeter (ABL 5 and OSM 3, Radiometer, Copenhagen, Denmark). C_a–v_O_2_ was calculated by standard formulae.

Cardiac output was calculated as VO_2_ divided by C_a–v_O_2_. DO_2_ was calculated as cardiac output multiplied by arterial O_2_ content.

We also calculated P_v–a_CO_2_ and P_v–a_CO_2_/C_a–v_O_2_. According to Fick’s principle, venoarterial CO_2_ content difference (C_v–a_CO_2_) was calculated as VCO_2_ divided by cardiac output.

### Experimental procedure

Basal measurements were taken after a period of no less than 30 min after systemic VO_2_ and VCO_2_ became stable. Animals were then assigned to hemodilution (*n* = 8) and hemorrhage (*n* = 8) groups. In the hemodilution group, we performed a stepwise hemodilution through isovolemic exchange of blood with 6% hydroxyethyl starch 130/0.4 in 0.9% NaCl (Voluven, Fresenius Kabi, Bad Homburg, Germany). The amount of blood exchanged to reach desired levels of hematocrit of about 0.15, 0.10, and 0.05 in each step was estimated as previously referred [12]. In the hemorrhage group, consecutive bleedings of 5–10 mL/kg were performed. Similar reductions in systemic VO_2_ were pursued in both groups in order to reach comparable degrees of anaerobic metabolism. Measurements were taken at 30, 60, and 90 min. Blood temperature was kept constant throughout the study with a heating lamp.

At the end of the experiment, the animals were killed with an additional dose of pentobarbital and a KCl bolus.

### Data analysis

Data were assessed for normality and expressed as mean ± SEM. Groups were compared with two-way repeated measures of ANOVA. After a *P* < 0.05 for time × group interaction, a post hoc Student’s *t* test with Bonferroni correction was used for pairwise comparisons. Simple linear regression analysis with P_v–a_CO_2_/C_a–v_O_2_ as the outcome variable was conducted, and variables showing a *P* value <0.20 or physiologically plausible were entered in a multiple linear regression model. The final model was tested for the presence of collinearity (VIF test). All analyses were done with Stata statistical software (Stata Corporation, Release 12, College Station, TX, USA).

## Results

In both groups, DO_2_ fell progressively. In the hemorrhage group, the decrease in DO_2_ was primarily related to the reduction in cardiac output from 166 ± 13 to 54 ± 6 mL/min/kg (*P* < 0.0001). In addition, Hb fell from 8.4 ± 0.5 to 6.6 ± 0.4 g/dL (*P* < 0.0001). In hemodilution group, the drop in DO_2_ was completely explained by the reduction in Hb from 8.3 ± 0.4 to 1.2 ± 0.1 g/dL (*P* < 0.0001). Cardiac output concurrently increased from 165 ± 16 to 373 ± 41 mL/min/kg (*P* < 0.0001).

**Fig. 1:**
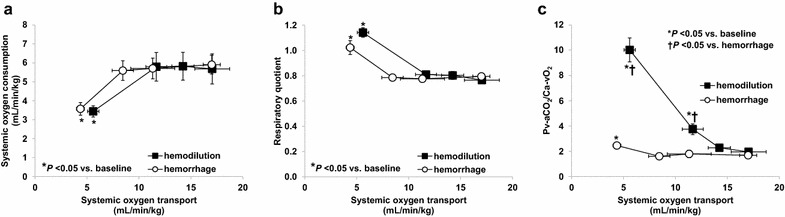
Relationship between oxygen transport to oxygen consumption (**a**), respiratory quotient (**b**), and venoarterial PCO_2_ difference-to-arteriovenous O_2_ content difference ratio (P_v–a_CO_2_/C_a–v_O_2_) (**c**). Oxygen consumption fell and respiratory quotient increased only in the last step of hemodilution and hemorrhage. In hemodilution, the increase in P_v–a_CO_2_/C_a–v_O_2_ was higher than in hemorrhage and appeared before the development of oxygen supply dependency

In the last stage, there were similar decreases in VO_2_ and increases in RQ in both groups. P_v–a_CO_2_/C_a–v_O_2_ also increased in the last stage in the hemorrhage group. P_v–a_CO_2_/C_a–v_O_2_ increased after the second step in the hemodilution group, and the increases were higher than in hemorrhage group (Fig. 1).

**Fig. 2:**
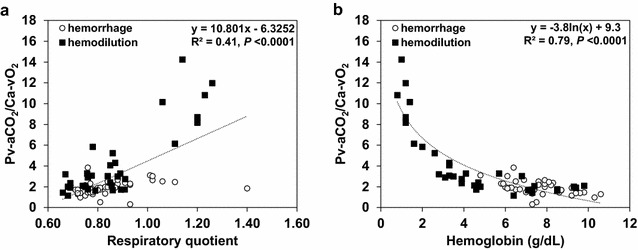
Correlation of venoarterial PCO_2_ difference-to-arteriovenous O_2_ content difference ratio (P_v–a_CO_2_/C_a–v_O_2_) with respiratory quotient (**a**) and Hb levels (**b**). The correlation between P_v–a_CO_2_/C_a–v_O_2_ and RQ was statistically significant but moderate. In contrast, P_v–a_CO_2_/C_a–v_O_2_ and Hb levels were strongly correlated

P_v–a_CO_2_/C_a–v_O_2_ was strongly correlated with Hb levels and moderately with RQ (Fig. 2). A similar behavior was observed in hemorrhage group (*R* ^2^ = 0.23, *P* < 0.002 and *R* ^2^ = 0.12, *P* < 0.03). A multiple linear regression model, developed with data from both groups, found Hb, RQ, base excess, and mixed venous oxygen saturation and PCO_2_ as P_v–a_CO_2_/C_a–v_O_2_ determinants (adjusted *R* ^2^ = 0.86, *P* < 0.000001). Hb was the explanatory variable with the highest independent contribution to the prediction (highest *t* ratio) (Table 1). The model did not exhibit collinearity.

**Table 1:** Multiple linear regression model for the ratio of venoarterial PCO_2_ to arteriovenous oxygen content difference (P_v–a_CO_2_/C_a–v_O_2_)

P_v–a_CO_2_/C_a–v_O_2_	Coefficient	Standard error	*t* ratio	*P* value	[95% confidence interval]
Ln hemoglobin (g/dL)	−3.60	0.26	−13.62	<0.000001	−4.13 −3.08
Respiratory quotient	2.43	1.03	2.35	<0.03	0.37 4.49
Base excess (mEq/L)	−0.06	0.03	−2.42	<0.02	−0.12 −0.01
Mixed venous O_2_ saturation (fraction)	0.03	0.01	3.90	<0.0003	0.01 0.04
Mixed venous PCO_2_ (mmHg)	0.15	0.03	4.58	<0.00003	0.08 0.21
Intercept	−0.98	1.72	−0.57	0.57	−4.40 2.44

**Fig. 3:**
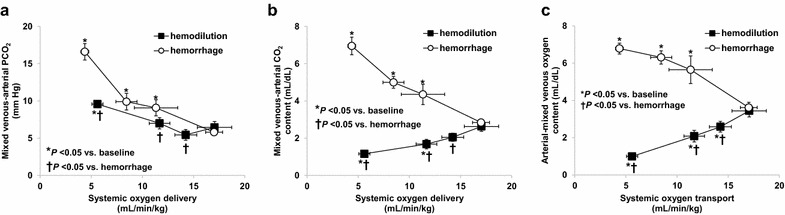
Relationship between oxygen transport to venoarterial PCO_2_ difference (P_v–a_CO_2_) (**a**), venoarterial CO_2_ content difference (C_v–a_CO_2_) (**b**), and arteriovenous O_2_ content difference (C_a–v_O_2_) (**c**). Hemodilution produced opposite effects on P_v–a_CO_2_ and C_v–a_CO_2_. C_v–a_CO_2_ decreased in hemodilution and increased in hemorrhage. These changes are the underlying explanation for different behavior of P_v–a_CO_2_/C_a–v_O_2_ in both groups

**Fig. 4:**
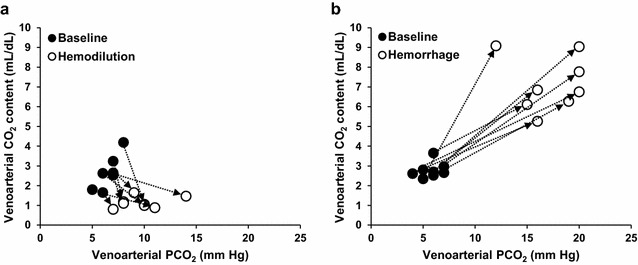
Changes in the relationship between venoarterial CO_2_ pressure and content differences in hemodilution (**a**) and hemorrhage (**b**). Hemodilution shifted CO_2_Hb dissociation curve to the *right*

P_v–a_CO_2_ increased in the hemorrhage group from the first stage and in hemodilution group only in the last phase. The increases in P_v–a_CO_2_ were higher in hemorrhage than in hemodilution, while C_v–a_CO_2_ increased in hemorrhage and decreased in hemodilution (Fig. 3). In the hemodilution group, there was a right shift in the relationship between CO_2_ pressures and contents (Fig. 4). During reductions in DO_2_, C_a–v_O_2_ increased in the hemorrhage group and fell in the hemodilution group (Fig. 3).

## Discussion

The main finding of this study was that P_v–a_CO_2_/C_a–v_O_2_ failed to properly reflect RQ in hemodilution. It increased before the appearance of the dependency of VO_2_ on DO_2_. Its correlation with RQ was moderate, but it showed a strong association with Hb levels. Indeed, P_v–a_CO_2_/C_a–v_O_2_ was more explained by Hb levels than by anaerobic metabolism. Changes in the dissociation of CO_2_ from Hb mostly account for these results.

Several studies have tried to link P_v–a_CO_2_/C_a–v_O_2_ with some events suggestive of anaerobic metabolism such as hyperlactatemia [4], decreased lactate clearance [5, 6], increased VO_2_ in response to fluid challenge [7, 8], and worse outcome [4]. Since RQ was not measured in those studies, it was not clear whether P_v–a_CO_2_/C_a–v_O_2_ effectively reflected the presence of anaerobic metabolism or was only the result of factors that could increase that ratio in the absence of anaerobic metabolism. In fact, P_v–a_CO_2_/C_a–v_O_2_ is not a straightforward variable. Although related to RQ, it might be hypothetically increased by several factors beyond anaerobic metabolism. Many of the changes in P_v–a_CO_2_/C_a–v_O_2_ might be ascribed to modifications of the CO_2_-Hb dissociation curve. Haldane effect, metabolic acidosis, and anemia can increase PCO_2_ for a given CCO_2_ [13]. In addition, taking into account the curvilinear characteristics of the dissociation curve, the effects are even greater at higher PCO_2_. When the slope of the dissociation curve flattens, substantial increases in P_v–a_CO_2_ may actually represent negligible increases in C_v–a_CO_2_. Therefore, high oxygen venous saturation [14], hyperlactatemia [15], and hemodilution [16] can increase P_v–a_CO_2_ even though C_v–a_CO_2_ remains unchanged.

In line with the previous discussion, our results showed that isovolemic anemia disproportionally increased P_v–a_CO_2_/C_a–v_O_2_, compared to hemorrhage. Furthermore, this ratio was elevated before the beginning of oxygen supply dependency. Progressive hemodilution was associated with opposing effects on P_v–a_CO_2_ and C_v–a_CO_2_: P_v–a_CO_2_ increased and C_v–a_CO_2_ decreased. Previous studies showed that decreasing hemoglobin levels results in widened P_v–a_CO_2_ for a given C_v–a_CO_2_ [16]. In a similar model of progressive hemodilution, the contrasting effects of low Hb levels on P_v–a_CO_2_ and C_v–a_CO_2_ were also noticed [10]. Therefore, increased P_v–a_CO_2_ is a predictable consequence of anemia.

Another expected consequence from hemodilution is the decrease in C_a–v_O_2_ [11]. Increases in oxygen extraction always occur in response to reductions in DO_2_, irrespective of the mechanism of oxygen supply limitation. The impact of the increase in oxygen extraction on C_a–v_O_2_, however, depends on cardiac output. According to Fick’s principle, C_a–v_O_2_ should widen in conditions of low cardiac output and decreased in states of reduced DO_2_ with increased cardiac output, if VO_2_ remains constant. Our study also confirmed this assumption.

As a result of the opposite effects of hemodilution on P_v–a_CO_2_ and C_a–v_O_2_, the ratio between both variables markedly augmented in the absence of anaerobic metabolism. The increase in P_v–a_CO_2_/C_a–v_O_2_ was even higher during the oxygen supply dependency, due to the interplay of the aforementioned factors and the ongoing anaerobic CO_2_ production.

Considering the coefficient of determination of the regression (*R* ^2^ = 0.41), RQ only explains a minor part of the P_v–a_CO_2_/C_a–v_O_2_ variability. As supported by the results of the multiple linear regression model, P_v–a_CO_2_/C_a–v_O_2_ is a complex variable that has several determinants. Although Hb was the main contributor to the prediction of P_v–a_CO_2_/C_a–v_O_2_, it was also influenced by RQ and by the changes in the dissociation of CO_2_ from hemoglobin induced by metabolic acidosis and Haldane effect. These effects were magnified at the flattened portion of the CO_2_Hb dissociation curve as shown by the impact of mixed venous PCO_2_ in the model.

A study has proposed a P_v–a_CO_2_/C_a–v_O_2_ cutoff of 1.4 for the identification of anaerobic metabolism [4]. This suggestion, however, should be carefully interpreted. The development of anaerobic metabolism is identified by acute increases in RQ, not by isolated values [1–3]. Actually, the normal range of RQ is 0.67–1.30 [17] depending also on other factors such as energy source [18] and overfeeding [19]. In our experiments, values of P_v–a_CO_2_/C_a–v_O_2_ during oxygen supply dependency were considerably higher (10.0 ± 2.7 and 2.5 ± 0.4 in hemodilution and hemorrhage groups, respectively).

Our findings do not challenge the value of P_v–a_CO_2_/C_a–v_O_2_ as an outcome predictor of critically ill patients, which was previously described [4]. The composite characteristics of P_v–a_CO_2_/C_a–v_O_2_, however, suggest that the prognostic ability might be mainly related to the interaction of several mechanisms, not only to anaerobic metabolism.

Our study has certain drawbacks. Secondary analyses pose inherent limitations that have been subject to critiques [20]. In addition, part of our analysis was based on calculations of CCO_2_, not in actual measurements [21]. This last procedure is complex and cumbersome and is not available in our laboratory. Accordingly, we calculated CCO_2_ from Fick’s principle. We prefer this method, because the different algorithms for computing CCO_2_ from blood gases and Hb are frequently misleading and can produce negative C_v–a_CO_2_ values. Finally, the experimental model of hemorrhage and hemodilution does not address the applicability of our results to septic conditions.

## Conclusions

Hemodilution produced higher increases in P_v–a_CO_2_/C_a–v_O_2_, compared to hemorrhage, and this ratio was widened even in the absence of oxygen supply dependency. These findings were related to the effects of anemia on CO_2_Hb dissociation curve and C_a–v_O_2_. Our results suggest that P_v–a_CO_2_/C_a–v_O_2_ is a multifactorial variable, which results from interactions among anaerobic metabolism, anemia, metabolic acidosis, and Haldane effect. Since it is not an accurate surrogate for RQ, values of P_v–a_CO_2_/C_a–v_O_2_ should be cautiously interpreted. Further studies in septic models are needed to confirm the limitations of P_v–a_CO_2_/C_a–v_O_2_ in such condition.

## Abbreviations

DO_2_: oxygen transport
VO_2_: oxygen consumption
RQ: respiratory quotient
VCO_2_: carbon dioxide production
P_v–a_CO_2_: venoarterial PCO_2_
C_a–v_O_2_: arteriovenous oxygen content difference
P_v–a_CO_2_/C_a–v_O_2_: ratio of venoarterial PCO_2_ to arteriovenous oxygen content difference
C_v–a_CO_2_: venoarterial CO_2_ content difference
